# Preface to the First Issue, JCDR

**DOI:** 10.4103/0975-3583.59976

**Published:** 2010

**Authors:** Zhou Peng

**Affiliations:** *Today’s Science, Tomorrow’s Health. JCDR, a platform for your expertise. North Carolina, USA December 16, 2009 Zhou Peng M.D. & Ph.D. Editor-in-Chief Winston-Salem*

In 1628, William Harvey published his epoch-making treatise “On The Motion Of The Heart And Blood In Animals”. This 70-page booklet laid the foundation for contemporary cardiology. Since then, life sciences research has not only facilitated the vigorous advances associated with the Industrial Technology Revolution Tidal Wave and the Information Technology Revolution Tidal Waves, but also paved the way for progress in the diagnosis and treatment of cardiovascular diseases (CVD). Time witnessed the following momentous milestones in cardiovascular medicine:

1. The clinical application of aspirin and other anti-thrombocyte, cardiotonics, diuretics, angiotensin converting enzyme inhibitor (ACEI), beta blocker and statin class lipid-lowering medications has significantly reduced the risk of the CVD patients, greatly improved the symptoms and the quality of lives and distinctly ameliorated the prognosis of CVD patients.
2. Implementation of accessory examination tools, including the stethoscope, electrocardiograph (ECG), modern high energy X-ray machines, echocardiography (Echo), nuclide scan, computerized tomography (CT), magnetic resonance imaging (MRI), and positron emission tomography (PET) has greatly enhanced our clinical diagnosis level of CVD.
3. The appearance of the pacemaker, implantable converter defibrillator (ICD), coronary angiography (CAG), stent and the drug-eluting stent, and the emergence of radio frequency catheter ablation (RFCA) and percutaneous coronary intervention (PCI) technology have led us into a new era of Interventional Cardiology.
4. Cardiopulmonary bypass technology, coronary artery bypass grafting (CABG) technology, heart transplant technology, off pump-bypass technology and the new generation of artificial heart development have all contributed to the improved precision of cardiac surgery techniques.

Building on the brilliant achievements of the Industrial Revolution and Information Technology Revolution Tidal Waves, we are moving towards the new era of a Life Science Technology Revolution Tidal Wave.

Today, cardiology is advancing in genotype – phenotype association of cardiovascular diseases, the structure and function of ion channels, the upstream and downstream mediators of signal transduction pathways, and the regulation of myocyte proliferation and apoptosis.

The elucidation of the nitric oxide (NO) signal transduction pathway and the determination of the potassium channel structure led to the recent choices of Nobel Prize laureates.

Stem Cell therapy is emerging as a hot topic in all kinds of cardiovascular disease congresses all over the world.

However, we are far from conquering the CVD. We are still faced with a more and more serious situation.

Because of the dramatic changes in lifestyle, increases in the aging population and because of striking changes in disease spectrum, since 1900, the morbidity and mortality rates of CVD have steadily increased at an alarming rate in the world. At the beginning of the 20th century, CVD accounted for less than 10 percent of all deaths worldwide. At its end, CVD accounted for nearly half of all deaths in the developed world and 25 percent in the developing world.^1^

Today, both the developed countries and the developing countries all over the world are challenged by the prevalence of CVD.

In the United States only, an estimated 80.7 million adults (one in three) have one or more types of CVD. CVD is the leading killer of both men and women among all racial and ethnic groups and accounted for 34.2% (829,072) of all 2,425,900 deaths in 2006, or 1 of every 2.9 deaths.^2^

In China, where the people account for one fifth of the world’s population, since the 1950s, the mortality of CVD increased threefold as a percentage of total deaths, from 12% to 36%.^3^

In India, where the people account for one sixth of the world’s population, the estimated data suggests that CVD accounts for 24% of total deaths.^4^

More seriously, a series of controllable CVD risk factors, including smoking, hypertension, fat consumption, high plasma cholesterol levels, physical inactivity, diabetes mellitus and obesity are spreading at an unprecedented speed all over the world.

CVD will dominate as the major cause of death by 2020, accounting for at least one in every three deaths all over the world.^1^

It’s estimated that by 2020, we could meet the pinnacle of CVD.^1^

CVD also presents a heavy burden to family and society, in terms of both medical costs and human suffering. In 2008 alone, the estimated direct and indirect cost of CVD only in the United States was $448.5 billion, an alarming statistic under these severe economic circumstances.^2^

Establishing a comprehensive, global, preventive and therapeutic line of defense against CVD spreading all over the world is a sacred duty for governments, doctors and basic research scientists in the field.

Further enhancement of basic and clinical CVD research is the critical responsibility incumbent on all of us, as diverse professionals in the field of CVD.

Based on the issues described herein, a group of outstanding cardiologists, basic research scientists from China, India, United States and Japan etc. have established this double-blinded, peer-reviewed, international professional journal, Journal of Cardiovascular Disease Research (JCDR) under the strong support of the non-profit, medical magazine running and the experienced publisher, E-Manuscript.

JCDR covers basic scientific research to clinical practice and deals with all areas of cardiovascular disease. JCDR publishes articles on work at the molecular, subcellular, cellular, tissular, organic and systemic levels with an emphasis on the pathophysiological mechanisms of CVD, while JCDR appreciates articles related to prevention, clinical observation, medication, interventional therapy, cardiac surgery and nursing. Cardiovascular research scientists, physicians, surgeons, pharmacists, occupational therapists, health care professionals and other related experts in cardiovascular disease research field are the intended directors, actors and audiences of JCDR.

JCDR is a platform, a platform for international academic information exchange among professionals in the field of CVD community all over the world.

JCDR is a platform, a platform for presentation and participation of your clinical or basic research achievements.

JCDR is a platform, a platform for revelation of your intellectual sparks.

It is my sincere hope that you will dedicate your spark of wisdom to this JCDR platform. Perhaps, your spark will ignite into a bright and sustaining flame to illuminate your other colleagues.

With the New Year’s bell striking, here, on behalf of our editorial board, I am pleased to announce the launch of Journal of Cardiovascular Disease Research (JCDR), a new non-profit official journal published by E- Manuscript.
